# Supplementary material for: An analysis of factors that influence personal exposure to toluene and xylene in residents of Athens, Greece
Source: BMC Public Health. 2006 Feb 28;6:50. doi: 10.1186/1471-2458-6-50 (PMC1434731; doi:10.1186/1471-2458-6-50)
Supplement: Additional File 1 — Sample contains Tables 1, 2and 3 Table 1 shows house characteristics in relation to annual (median) toluene and xylene measurements ( μg/m3) Table 2 shows correlation coefficients between personal exposure and indoor residential concentration during the six monitoring campaigns Table 3 shows Benzene:Toluene:Xylene (BTX) ratios during the measurement periods [file 1471-2458-6-50-S1.doc]

## Additional file 1

## Table 1 - Characteristics of volunteers’ houses and corresponding annual (median) toluene and xylene measurements, personal exposures and indoor concentrations

|  | Toluene (μg/m^3^) | Xylene (μg/m^3^) |
| --- | --- | --- |

|  | n % | Personal | Home | Personal | Home |
| --- | --- | --- | --- | --- | --- |
| Type & floor of house  > 4^th^  2^nd^ – 3^rd^  ground – 1^st^ floor  detached house | 16 32  13 26  14 28  7 14 | 52.5  60.6  71.2  63.8 | 39.6  39.0  38.9  31.4 | 59.0  71.0  75.1  63.4 | 36.7  40.2  35.0  27.5 |
| Heating mode  Fireplace  Oil oven  Natural gas oven  Central heating | 5 10  2 4  4 8  39 78 | 60.8  74.9  73.7  60.5 | 31.2  39.9  42.9  39.3 | 54.9  88.0  78.0  66.0 | 26.1  40.8  38.2  36.9 |

## Table 2 - Correlation coefficients (r) between personal exposure and indoor residential concentration during the six monitoring campaigns

|  | | Toluene | Xylene |
| --- | --- | --- | --- |
| PERIOD | 1^st^ – SEP | 0.14 | 0.02 |
|  | 2^nd^ – DEC | 0.31 | 0.09 |
|  | 3^rd^ – FEB | 0.52 | 0.23 |
|  | 4^th^ – APR | 0.29 | 0.14 |
|  | 5^th^ – JUN | 0.11 | 0.06 |
|  | 6^th^ – SEP | 0.68 | 0.42 |

## Table 3 - Benzene:Toluene:Xylene (BTX) ratios during measurements periods

|  | Urban levels | Personal exposures | Indoor levels |
| --- | --- | --- | --- |
| 1^st^ - Sep | 1:3.2:3.4 | 1:3.4:3.6 | 1:3.8:3.6 |
| 2^nd^ - Dec | 1:3.4:3.7 | 1:3.4:3.5 | 1:3.7:3.5 |
| 3^rd^ - Feb | 1:3.5:4.1 | 1:3.7:3.8 | 1:3.9:3.8 |
| 4^th^ - Apr | 1:3.4:3.9 | 1:4.1:4.4 | 1:4.1:4.1 |
| 5^th^ - Jun | 1:3.5:4.0 | 1:4.3:4.5 | 1:5.6:5.1 |
| 6^th^ - Sep | 1:3.5:3.8 | 1:3.5:3.8 | 1:4.2:4.1 |
| Annual | 1:3.4:3.9 | 1:3.6:3.9 | 1:4.5:4.2 |
